# Potential molecular targets and drugs for basement membranes-related intervertebral disk degeneration through bioinformatics analysis and molecular docking

**DOI:** 10.1186/s12891-023-06891-z

**Published:** 2023-10-02

**Authors:** Zelin Zhou, Weicheng Qin, Peng Zhang, Jiahui He, Zhaojun Cheng, Yan Gong, Guangye Zhu, De Liang, Hui Ren, Xiaobing Jiang, Yuping Sun

**Affiliations:** 1 The First Clinical Medical College, Guangzhou University of Chinese Medicine, Guangzhou, Guangdong P.R. China; 2 Department of Spinal Surgery, The First Affiliated Hospital of Guangzhou University of Chinese Medicine, Guangzhou, Guangdong P.R. China; 3 Pingshan General Hospital, Southern Medical University, Shenzhen, Guangdong P.R. China; 4 Pingshan District People’s Hospital of Shenzhen, Shenzhen, Guangdong P.R. China; 5 Rehabilitation Department, Pingshan District People’s Hospital, Shenzhen, P.R. China

**Keywords:** Basement membranes, Annulus fibrosus, Intervertebral disc degeneration, Bioinformatic analysis, Molecular docking

## Abstract

**Background:**

Through bioinformatics analysis to identify the hub genes of Intervertebral disc degeneration (IVDD) associated with basement membranes (BMs) and find out the potential molecular targets and drugs for BMs-related annulus fibrosus (AF) degeneration based on bioinformatic analysis and molecular approach.

**Methods:**

Intervertebral disc degeneration (IVDD) related targets were obtained from GeneCards, DisGenet and OMIM databases. BMs related genes were obtained from Basement membraneBASE database. The intersection targets were identified and subjected to protein-to-protein interaction (PPI) construction via STRING. Hub genes were identified and conducted Gene ontology (GO) and pathway enrichment analysis through MCODE and Clue GO in Cytospace respectively. DSigDB database was retrieved to predict therapeutic drugs and molecular docking was performed through PyMOL, AutoDock 1.5.6 to verify the binding energy between the drug and the different expressed hub genes. Finally, GSE70362 from GEO database was obtained to verify the different expression and correlation of each hub gene for AF degeneration.

**Results:**

We identified 41 intersection genes between 3 disease targets databases and Basement membraneBASE database. PPI network revealed 25 hub genes and they were mainly enriched in GO terms relating to glycosaminoglycan catabolic process, the TGF-β signaling pathway. 4 core targets were found to be significant via comparison of microarray samples and they showed strong correlation. The molecular docking results showed that the core targets have strong binding energy with predicting drugs including chitosamine and retinoic acid.

**Conclusions:**

In this study, we identified hub genes, pathways, potential targets, and drugs for treatment in BMs-related AF degeneration and IVDD.

## Background

Intervertebral disc degeneration (IVDD) is a leading cause of low back pain, which significantly impacts patients’ quality of life and poses a significant burden on the economy [1, 2]. The annulus fibrosus (AF), a critical component of the intervertebral disc (IVD), encloses the nucleus pulposus (NP) and collaboratively accommodates mechanical loads, providing stability to the spinal column. Notably, IVDD arises due to an exploitation of the structural integrity of the AF before disc degeneration occurs [3]. Although the mechanisms underlying IVDD are not fully understood, an imbalance of anabolic processes in the AF may result in decreased tensile resistance, leading to IVDD [4]. Reduced abundance and quality of extracellular matrix (ECM) are hallmark features of disc degeneration [5]. Basement membranes (BMs) are a type of ECM that form sheet-like structures surrounding most tissues, including the IVD [6, 7]. Melrose et al. [8] used immunolocalization patterns to observe the developing human fetal spine and found that perlecan, the multidomin heparan sulfate proteoglycan of BMs, is a major component of the cartilage primordium in the developing human fetal spine. In their study, aggrecan staining was generally stronger in AF and heparan sulfate was diffusely distributed in the outer AF. Several studies [9, 10] have conducted bioinformatic analyses to investigate the relevant genes and pathways associated with IVDD. However, these studies did not explore the potential role of BM-related molecules in the pathogenesis of IVDD. In addition, AF may serve as a target tissue for the treatment of IVDD. Therapies based on the use of biological molecules, cell-based therapies, gene therapies and tissue engineering are applied in AF in recent decades [11]. Despite extensive research on the pathogenesis of IVDD, there is currently a lack of bioinformatics studies focused on drug prediction in the context of AF repair and regeneration. Therefore, it is imperative to gain a comprehensive understanding of the biological mechanisms underlying IVDD and the molecules associated with its BM, as this knowledge may facilitate the identification of novel therapeutic targets and drugs for AF repair and regeneration.

**Fig. 1:**
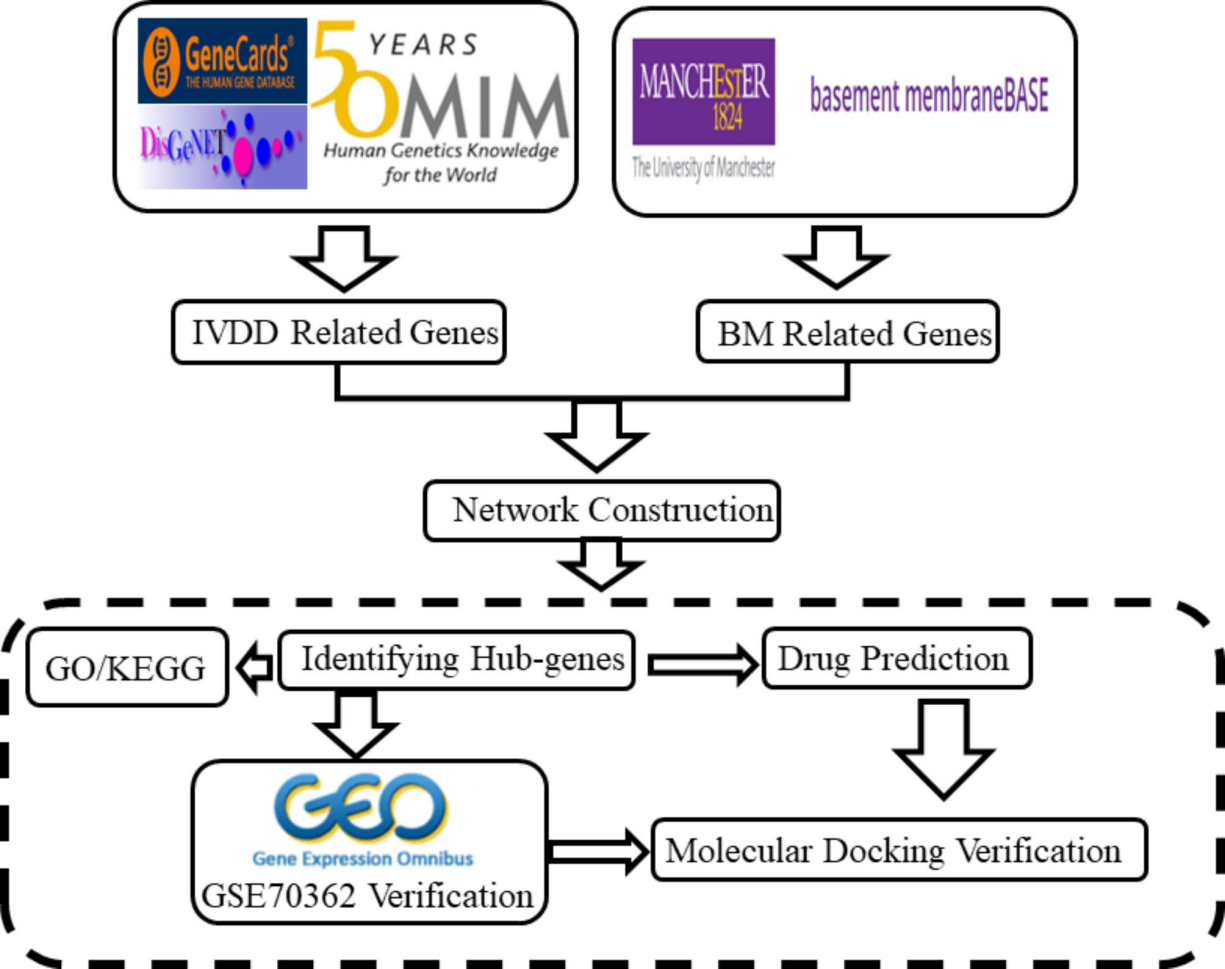
The flow chart of this study. IVDD = intervertebral disc degeneration; BM = basement membrane; GO = Gene Ontology; KEGG = Kyoto Encyclopedia of Genes and Genomes

In this study, we aimed to identify genes associated with IVDD and BMs by conducting an extensive search of multiple disease databases and the latest BMs database [7]. We then constructed a protein-to-protein interaction (PPI) network and identified hub genes. Gene ontology (GO) and pathways enrichment analysis were performed to gain a deeper understanding of the functional roles of these genes. To further validate our findings, we utilized transcriptome sequencing gene data from the Gene Expression Omnibus (GEO) database to verify the hub genes and combined the Drug Signature database (DSigDB) to predict potential therapeutic targets for AF. Finally, we conducted molecular docking to validate the interactions between the predicting drugs and targets. A flowchart detailing the methodology of our study is presented in Fig. 1.

## Methods

### Retrieving data

The keyword “intervertebral disc degeneration” was searched in GeneCards database (https://www.genecards.org), DisGeNet (http://www.disgenet.org), and the Online Mendelian Inheritance in Man (OMIM) (http://omim.org) to obtain the disease targets. BMs related genes were obtained from Basement membraneBASE (https://bmbase.manchester.ac.uk/). The R software (version 4.2.0) was used to identify intersection targets associated with IVDD and BMs through Venn diagram.

### PPI analysis and hub genes screening

The PPI network was constructed using the Search Tool for the Retrieval of Interacting Genes (STRING) (http://string-db.org) with a high confidence score ≥ 0.7. The PPI network was built based on the interaction data between the diseases targets of IVDD and BMs. The node were scored by the innate algorithm of the STRING database. Cytoscape software (version 3.8.0) were used to visualize. Hub genes were selected by using the plug-in molecular complex detection technology (MCODE). The parameter of MCODE is as follow: K-score = 2, degree cutoff = 2, max depth = 100, and node score cutoff = 0.2.

### Enrichment analysis

To further elucidate the biological functions if the hub genes, the Clue GO plug-in from the cytoscape software to conduct GO annotation and KEGG pathway analysis. The GO annotation focused on the biological processes relevant to our study. The results of the enrichment were filtered based on their *P*-value, with a *P*-value < 0.05 deemed statistically significant. The biological processes obtained were fused and clustered using ClueGo to provide a better understanding of the results. The KEGG was utilized to identify common pathways and maps, as well as to determine potential associations between key genes and pathways, and between pathways themselves. This approach can provide valuable insights for future research and aid in the development of novel therapeutic approaches for a range of diseases and conditions.

### Drug prediction and molecular docking

DsigDB database (http://dsigdb.tanlab.org) was utilized to analyze the hub genes and identified potential drug candidates. To investigate the potential docking mode and binding energy between targets and drugs, molecular docking was performed by using AutoDock software (version 1.5.6). The structures of critical targets were retrieved from RCSB Protein Data Bank (http://www.rcsb.org/) in PDB format. The 3D structures of candidate drugs were obtained from PubChem (http://pubchem.ncbi.nlm.nih.gov), which were transformed by Open Babel Toolkit (version 2.4.1) into a MOL2 file format. The AutoDock software was used to optimize proteins’ structure for molecular docking. Finally, the PyMOL software (version 2.4.1) was conducted for the visualization of docking results.

### Hub genes verification and correlation analysis

Microarray dataset GSE70362 was downloaded from the GEO database (http://www.ncbi.nlm.nih.gov/geo/). According to the Thompson grading of the fibrous ring tissue samples, those with Thompson grade 1–2 were defined as the control group, and those with Thompson grade 3–5 were defined as the degeneration group. There were 8 samples in the control group and 12 samples in the degeneration group. Furthermore, the expression of hub genes was verified in GSE70362. The comparison between the expression of hub genes from the dataset was conducted with the student t-test. After that, the correlation of the significant genes was analyzed by Spearman correlation analysis. *P*-value < 0.05 was considered significant.

## Results

### Intersection targets of IVDD and BMs

**Fig. 2:**
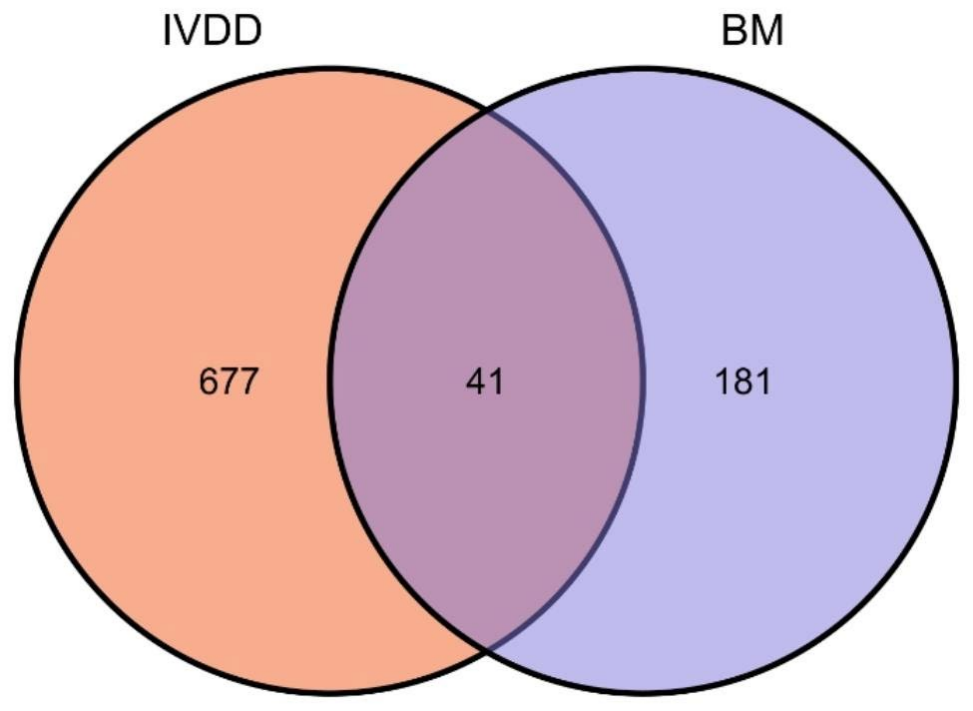
The Venn diagram. IVDD: Intervertebral disc degeneration. BM: Basement membrane

After eliminating duplicate genes, we obtained a total of 718 IVDD-related genes from reliable sources such as GeneCards, DisGeNet, and OMIM. Additionally, we retrieved 222 genes associated with BMs-related conditions from the Basement membraneBASE database. By intersecting these two gene sets, we identified 41 overlapping targets that were both IVDD-related and associated with BMs (Fig. 2).

### PPI network and module analysis

**Fig. 3:**
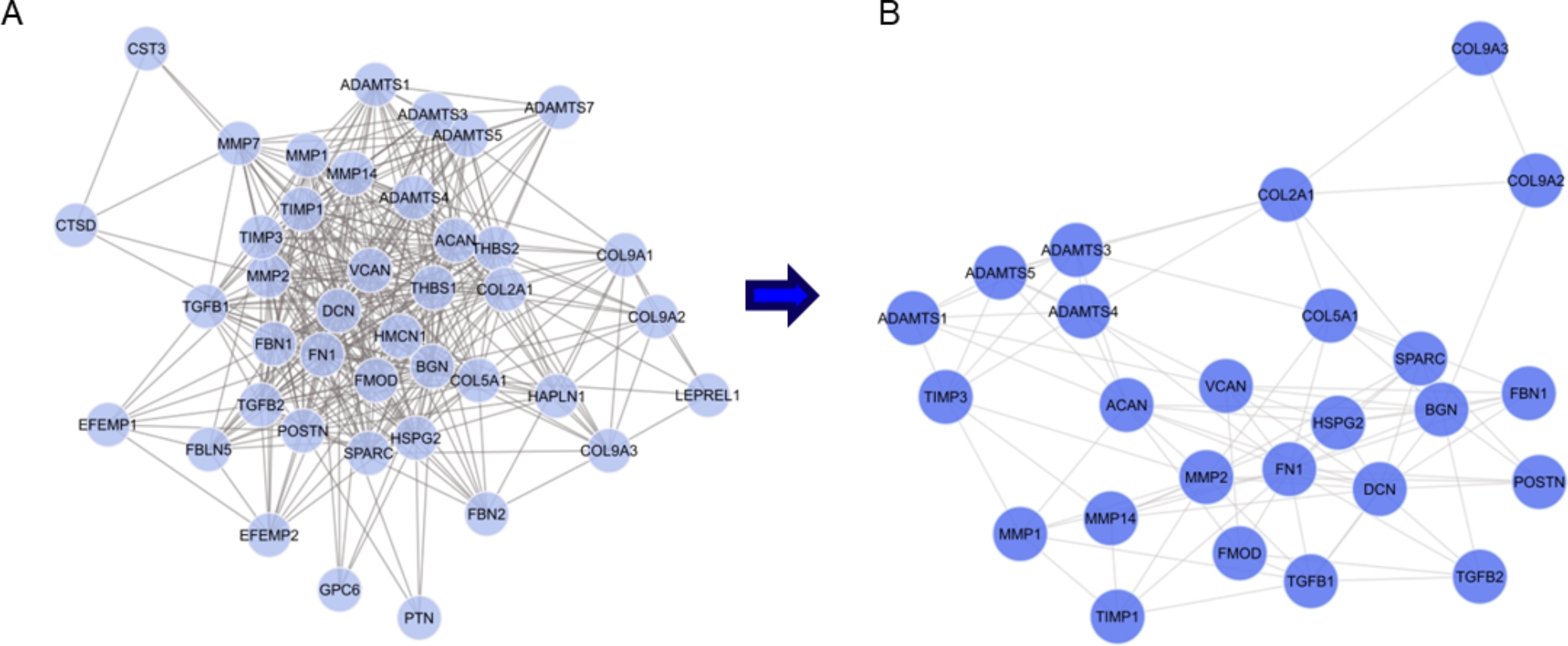
**(A)** Protein and protein interaction (PPI) network according to intersection targets. **(B)** The 25 hub genes screened by MCODE plug-in

The PPI network was constructed using STRING, consisting of 41 nodes and 381 edges, illustrating the connections between each protein (Fig. 3A). The average node degree was 18.6, indicating a significant level of interaction among these proteins, and the average local clustering coefficient was 0.741, reflecting a satisfactory level of clustering within the network. To identify key proteins within the network, we applied the MCODE plug-in and identified 25 hub genes. Subsequently, we constructed a separate PPI network focusing specifically on these 25 hub genes (Fig. 3B). This network comprised 25 nodes and 103 edges, with an average node degree of 7.63 and an average local clustering coefficient of 0.491. The increased number of edges in the network signifies a greater degree of interconnectedness and highlights the importance of the identified targets within the network.

### Enrichment analysis of hub genes

**Fig. 4:**
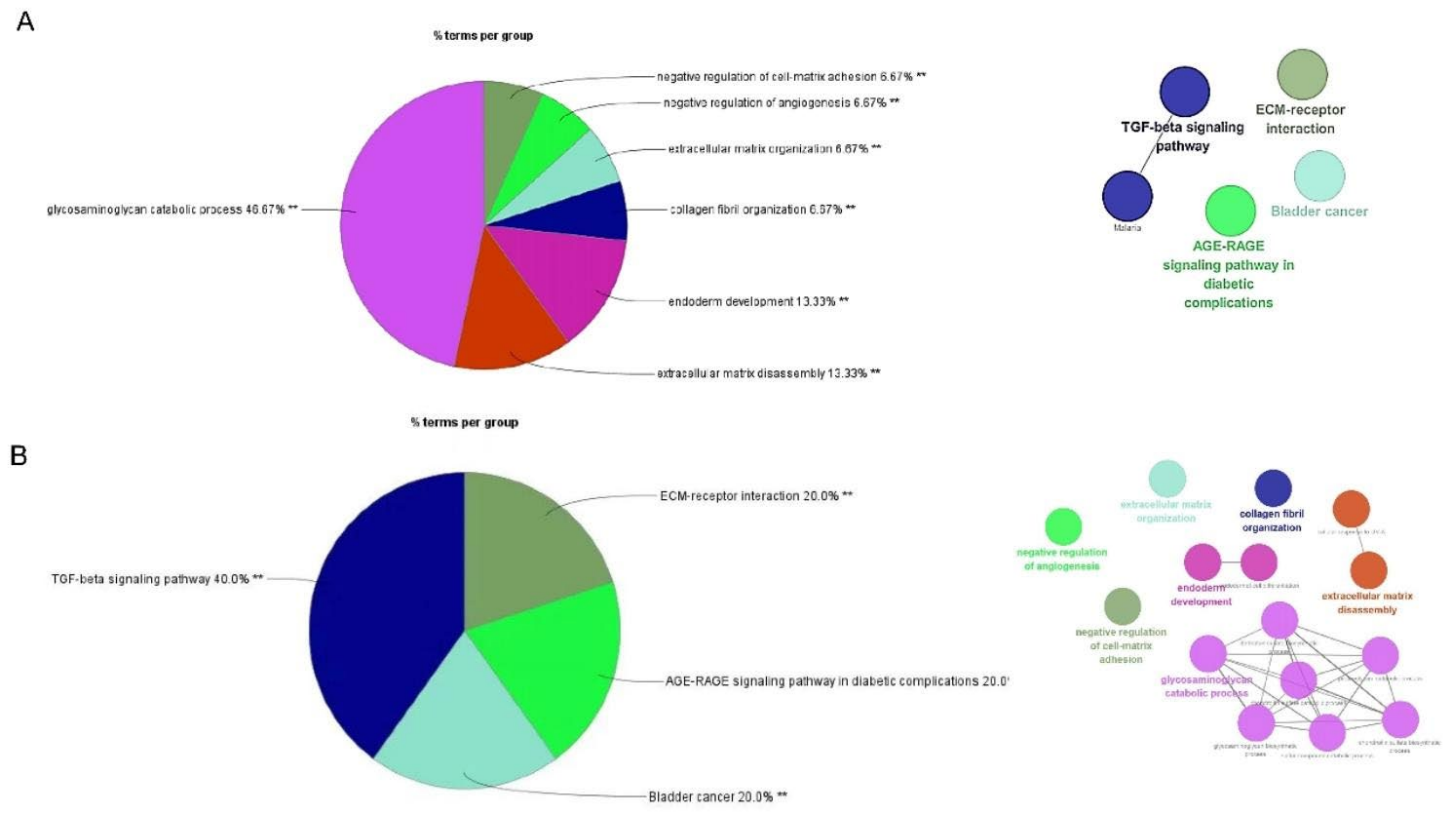
**(A)** Gene Ontology (GO) enrichment according to intersection targets of IVDD and BMs. The pie chart on the left represents the rate of pathways enrichment. (B) Pathway enrichment according to intersection targets of IVDD and BMs. The pie chart on the left represents the rate of enrichment. The network chart on the left represents the connection of biology processes and pathways. ^**^P-value < 0.01

We utilized Clue GO for GO annotation and KEGG pathway analysis of the previously identified hub genes. By applying a significance threshold of *P*-value < 0.05, we identified the significantly enriched GO terms, as shown in Fig. 4. Our analysis revealed that these hub genes, associated with both IVDD and BMs-related conditions, were primarily involved in various biological processes. These processes included glycosaminoglycan (GAG) catabolic process, extracellular matrix (ECM) disassembly, endoderm development, collagen fibril organization, ECM organization, negative regulation of angiogenesis, and negative regulation of cell-matrix adhesion (Fig. 4A). The focus of these biological processes predominantly centered around the synthesis and metabolic functions of substances such as ECM and GAG. In terms of KEGG pathway analysis, the major pathways enriched by these hub genes included the TGF-β signaling pathway, ECM-receptor interaction, AGE-RAGE signaling pathway in diabetic complications, and bladder cancer (Fig. 4B). Notably, the TGF-β pathway, which is closely linked to ECM, and the receptor-related pathway of ECM, were prominently enriched, aligning with the results obtained from the GO analysis.

### Prediction of potential drugs and molecular docking results

**Fig. 5:**
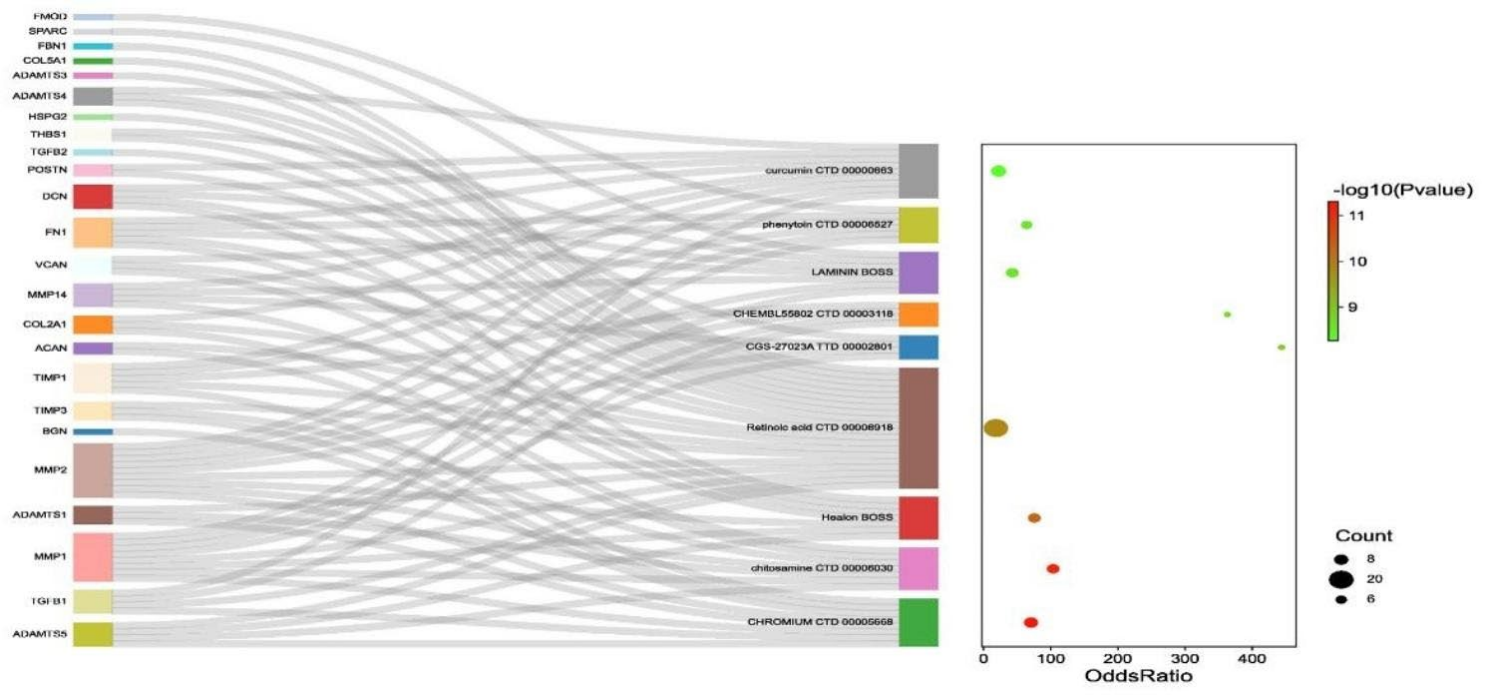
Sankey diagram presented the relationship with hub genes and predicted drugs. The right is the bubble diagram of gene enrichment. The gradual color represents the P-value and the increased bubble size represents the gene count

**Fig. 6:**
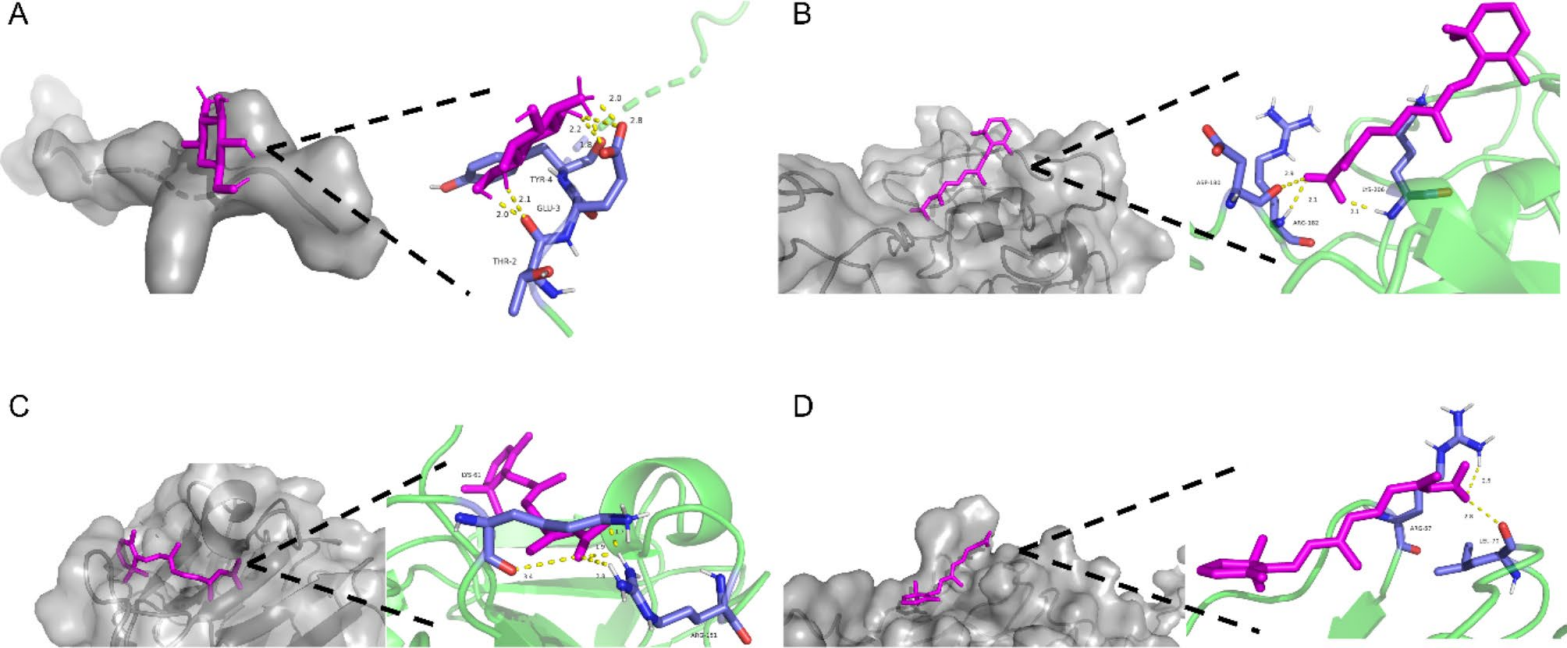
Molecular docking diagram of candidate drugs to target. **(A)** ACAN-chitosamine; **(B)** FBN1-retonoic acid; **(C)** HSPG2-retonoic acid; **(D)** DCN-retonoic acid

Using the DSigDB database for prediction, we identified the top 5 predicted drugs for preventing AF degeneration based on p-value: Chromium, Chitosamine, Healon, Retinoic acid, and CGS-27,023 A. We then considered the safety of these drugs in humans and their biological activity to select candidates for further study in the context of both MBs-related AF degeneration and IVDD. Among the selected drugs, Chitosamine and Retinoic acid emerged as promising candidates. We investigated their potential interaction with critical genes involved in both MBs-related AF degeneration and IVDD, including ACAN, DCN, FBN1, and HSPG2 (Fig. 5). Notably, our analysis revealed that Chitosamine displayed binding energy of -4.52 kcal/mol with ACAN. In comparison, Retinoic acid exhibited binding energies of -6.45 kcal/mol, -8.62 kcal/mol, and − 5.88 kcal/mol with FBN1, HSPG2, and DCN, respectively (Fig. 6). By incorporating the identification of unique drug selections associated with both MBs-related AF degeneration and IVDD, our study highlights the potential therapeutic relevance of Chitosamine and Retinoic acid. These findings warrant further investigation into their effectiveness in mitigating both conditions and provide valuable insights into potential therapeutic targets for the development of future treatments.

### Hub genes verification and correlation analysis by GEO

**Fig. 7:**
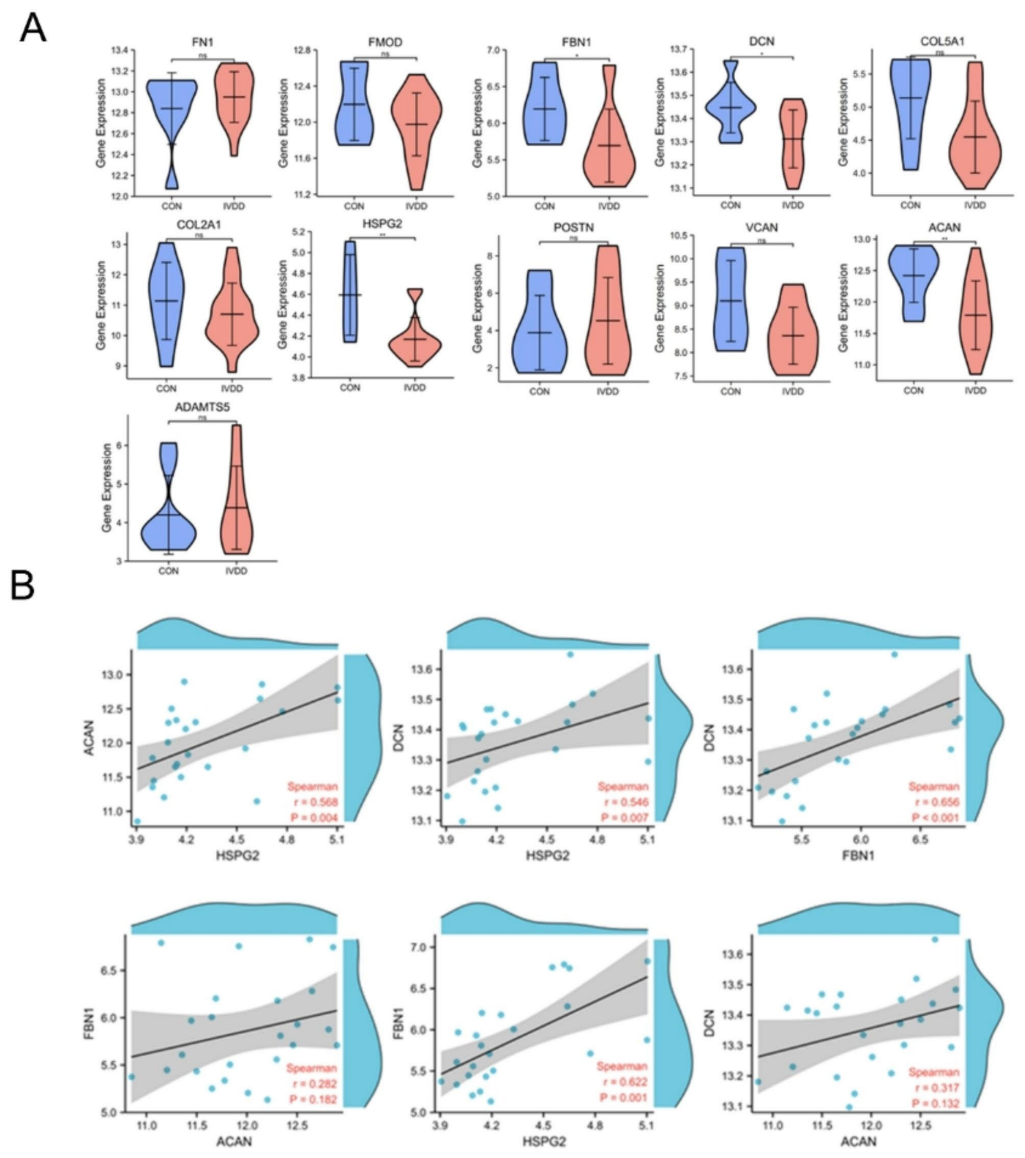
**(A)** Validation of hub genes in GSE70362. Con = healthy groups; IVDD = intervertebral disc degeneration group. ns represents no significant. ^*^P-value < 0.05; ^**^P-value < 0.01. **(B)** Correlation of hub genes with significantly different expression

We utilized samples from AF sources in the GSE70362 dataset to validate the hub genes. Among the 11 genes selected based on the PPI network and MCODE plug-in analysis, we found that ACAN, DCN, FBN1, and HSPG2 exhibited more significant differential expression (Fig. 7A). These hub genes demonstrated a more vital binding ability with the predicted drugs according to the previous molecular docking results. Furthermore, we conducted correlation analysis and observed that, except for FBN1 and ACAN, and DCN and ACAN, the expression of the remaining genes showed a positive correlation (Fig. 7B). This analysis provides additional evidence supporting the potential involvement of these hub genes in the context of AF degeneration and IVDD.

## Discussion

IVDD is a multifaceted disease related to the spine, and its progression cannot be reversed by current treatments. In healthy IVD tissue, the ECM provides a dynamic microenvironment that constantly undergoes remodeling, enabling it to withstand compression and torsion forces [12, 13]. BMs are a type of cell-adherent ECM composed of glycoproteins and proteoglycans, such as laminins and collagens, which are also abundant in IVD tissue, including the NP and AF [14, 15]. Despite limited research on BMs in IVDD, understanding the metabolic signature and relevant mechanisms of ECM is crucial for comprehending the cellular mechanisms of musculoskeletal degenerative diseases, including IVDD [12]. Therefore, it is reasonable to assume that the metabolic disorder of BMs and ECM may contribute to the pathogenesis of IVDD. However, the specific mechanisms of IVDD associated with BMs and ECM remain inconclusive. Consequently, using multiple databases, we identified a series of BMs-related genes to gain insight into the pathogenic mechanism of IVDD and explore potential drug candidates.

In this study, we conducted GO and pathway enrichment analyses in order to identify key biological processes and signaling pathways involved in IVDD. The essential biological processes are related to GAG catabolic, ECM disassembly, endoderm development, collagen fibril organization, and ECM organization. Of particular importance is the role of GAG catabolism, which has been shown to play a crucial role in human IVD. Proteoglycans, which contain GAGs, are degraded and depleted during IVDD, leading to the loss of ECM integrity [16]. Aberrant changes in GAG chain substitutions during degeneration can exacerbate this process [17]. To maintain normal IVD function, it is essential to maintain high concentrations and charges of proteoglycans, with functional proteoglycans possessing as many sulfated GAG chain substitutions as possible. However, enzymes like XT-1 and GlcAT-I for GAG biosynthesis are downregulated with age and degeneration, leading to diminished functional capacity of proteoglycans and abnormal GAG chain elongation [18, 19]. Additionally, our enrichment analysis revealed that the metabolism and regulation of ECM are also crucial in IVDD. As IVDD progresses, collagen type II and proteoglycans decrease notably, resulting in less resistance to loading of the ECM [20, 21]. Furthermore, the increase of fibrotic-like collagen type I contributes to the stiffness of IVD [22]. Therefore, a comprehensive understanding of the role of ECM in IVDD is essential for the development of novel treatments. Moreover, the disassembly and organization of the extracellular matrix (ECM) play a crucial role in the pathogenesis of IVDD with BMs, as revealed by the GO analysis. The ECM is a dynamic three-dimensional structure that appears in various tissues and can remodel and degenerate, contributing to the maturation and degeneration of the IVD [23, 24]. With the aging of the IVDD process, impaired ECM organization occurs simultaneously. In the worsened microenvironment, degenerative IVD tissue significantly affects the expression of ECM proteins. Razaq et al. [25] found that the synthesis of ECM proteins decreased in NP cells as the extracellular pH declined from 7.4 to 6.3. Furthermore, disassembled ECM has been detected in diseased IVDs. Patel et al. [26] demonstrated the presence of aggrecan fragmentation in human IVD tissue with disorders. The destruction of aggregating proteoglycans may be due to the failure of newly synthesized aggrecan to aggregate, the degradation of aggregating proteoglycans, and the dysfunction of link protein [27, 28].

The results of the pathway enrichment analysis indicate that the TGF-β signaling pathway is the primary pathway involved in IVDD. Previous studies have demonstrated a strong correlation between TGF-βs and TGF-β receptors [29]. Additionally, an animal study has suggested that the expression of TGF-βs and TGF-β receptors decreases in senescence-accelerated mouse models [30]. However, mRNA expression of TGF-βs and TGF-β receptors in both the NP and AF of older rabbits was found to be higher than that of younger rabbits [31]. The TGF-β signaling pathway is known to repair IVD degeneration by inhibiting ECM degradation and increasing ECM synthesis [32]. However, proinflammatory cytokines such as TNF-α and IL-1β can be secreted by immune cells and IVD cells, which promote ECM degradation by upregulating enzymes such as metalloprotease within thrombospondin motifs (ADAMTS), disintegrins, and MMPs [33]. Despite this, TGF-β has the ability to partially retard the upregulation of proinflammatory cytokines-induced matrix-degrading proteins by regulating the MAPK and NF-κB signaling pathways [34, 35]. Hu et al. [36] demonstrated that TGF-β can improve GAG biosynthesis through SMAD/3, RhoA/ROCK1, and MAPK pathways, which further highlights the importance of the TGF-β pathway in preventing IVD degeneration. In our study, we found that chitosamine and retinoic acid may have the potential to treat fibro-ring degeneration, and such an effect may be associated with the TGF-β pathway. This inspired us that future studies may need to focus on the role of drugs on this signaling pathway.

In this study, we identified four hub genes, namely ACAN, DCN, FBN1, and HSPG2, through the PPI network and validation of the GEO dataset. These hub genes exhibited significant differential expression between healthy and degenerated AF tissue. ACAN, also known as aggrecan, is a proteoglycan that creates an ion concentration imbalance between tissue and ambient fluid, resulting in an osmotic gradient for maintaining tissue hydration [37]. Previous studies have shown that the ECM of the inner AF is rich in aggrecan, which provides swelling pressure for necessary fiber elasticity and resistance against inner compressive, radical, and shear stresses [38–40]. Decorin (DCN) is an archetypal small leucine-rich proteoglycan (SLRP) that has been observed in lesioned AF tissue [41]. Previous studies have linked decorin to ECM damage [42] and remodeling and have detected it in damaged IVDs [34]. Zwambag et al. [43] demonstrated that decorin can bind to Toll-like receptor (TLR) 2/4 and improve pro-inflammatory chemokine and cytokine expression in AF cells. However, in the current study, decorin is differentially expressed in non-degenerative AF tissue. Therefore, future studies should investigate whether decorin expression changes with time course and mechanical properties.

Fibrillin-1 (FBN1) is a significant factor in the pathogenesis of Marfan syndrome, a genetic disorder characterized by connective tissue abnormalities [44]. In addition to its role in Marfan syndrome, Fibrillin-1 has been found to be involved in the regulation of the TGF-β signaling pathway, which controls the dispersal, storage, and release of ligands [45]. Multiple studies have demonstrated that Fibrillin-1 is widely distributed in human AF tissue and plays a crucial role in maintaining the homeostasis of the intervertebral disc bioenvironment [46, 47]. Heparin sulfate proteoglycan 2 (HSPG2) encodes for perlecan, a protein that is deposited in BMs and found in matrices in muscle, cartilage, and bone marrow. Hayes et al. [48] confirmed that Fibrillin-1 was differentially expressed in the HS-deficient HSPG mutant mouse, which highlights the role of perlecan in influencing the matrix organization.

**Fig. 8:**
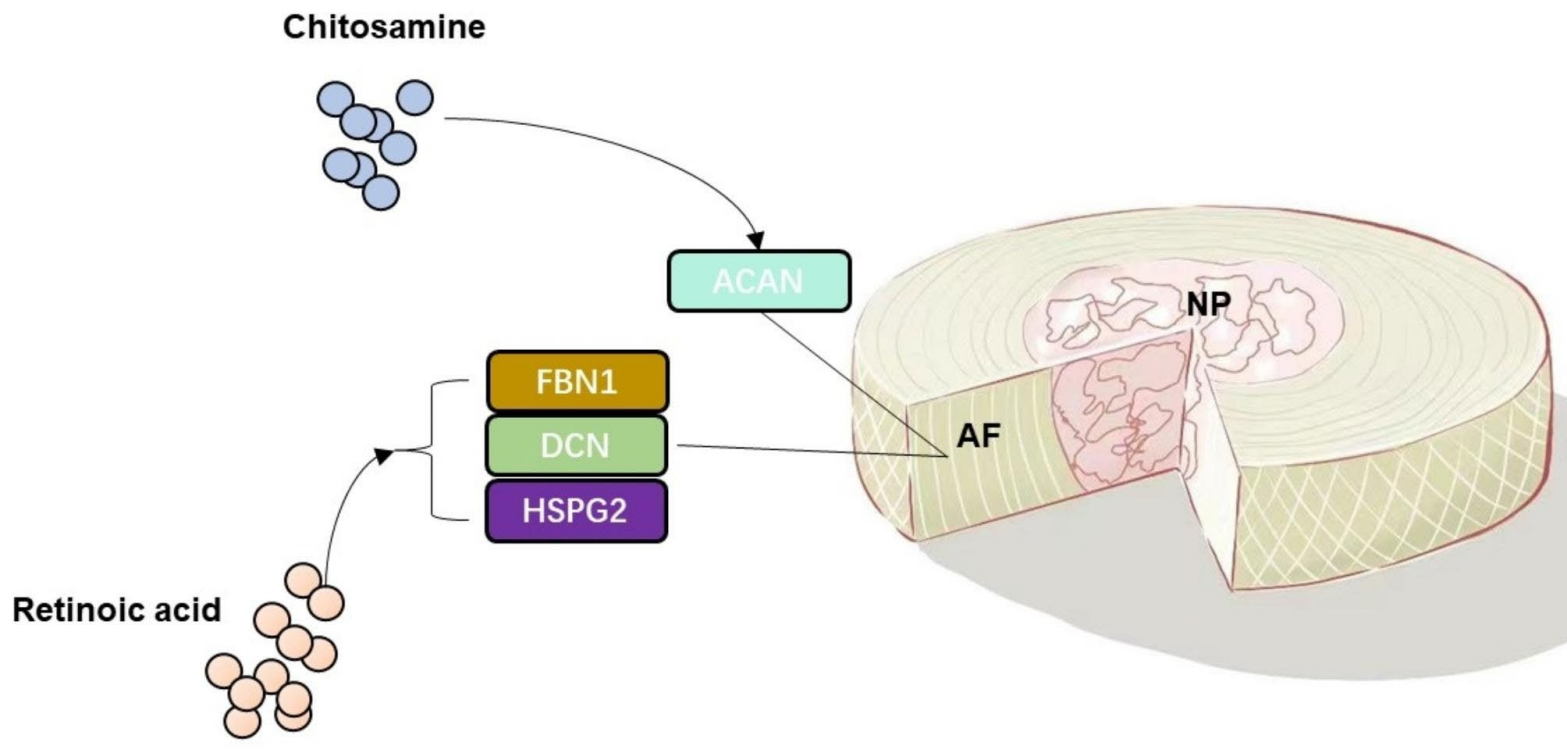
Schematic diagram of drug-target interaction. AF: annulus fibrosus; NP: nucleus pulposus

According to predictions from the DSigDB database, chitosamine and retinoic acid are significant drugs that target hub genes, including ACAN, DCN, FBN1, and HSPG2. To investigate the interaction of these proteins with the predicted drugs, molecular docking was performed for further validation. The results of docking indicate that chitosamine can bind well with ACAN and retinoic acid can bind well with DCN, FBN1, and HSPG2. Chitosamine, also known as glucosamine, is a naturally occurring component of glycosaminoglycans found in cartilage. It has been demonstrated to have a therapeutic effect on osteoarthritis [49]. However, it is essential to conduct further research to investigate the effect of glucosamine on AF tissue in vivo, under conditions of inflammatory and mechanical stimulation, to ensure its efficacy in living organisms. Previous studies have shown that the treatment of glucosamine could decrease the expression of aggrecan in rabbit AF cells [50]. Retinoic acid, an active metabolite of vitamin A, plays a crucial role in the modulation and maintenance of the immune response [51]. Despite its potential therapeutic application in treating AF degeneration, there have been few studies investigating the pharmacological effect of retinoic acid. Therefore, additional research is necessary to explore the potential molecular mechanism of retinoic acid and its predicted targets. A mechanism diagram of the pharmacologic interaction is presented in Fig. 8.

### Limitation

There are certain limitations inherent in our study: [1] Notably, not all of the hub genes identified in our analysis were present in the GSE70362 dataset, resulting in the inability to validate the expression of 14 genes [2]. While molecular docking provided valuable insights through computer simulations, it is crucial to acknowledge that this study did not experimentally verify the actual protein-drug molecule docking [3]. Further investigations are warranted in future studies to fully elucidate the specific molecular mechanisms underlying the action of drugs on AF.

## Conclusion

In conclusion, our results show that the metabolism of ECM and the TGF-β signaling pathways are important in the degeneration of IVD. BMs-related genes including ACAN, DCN, FBN1, and HSPG2 are the main targets for impeding the degeneration of AF tissue. Chitosamine and retinoic acid may be the potential therapeutic drugs for treating IVDD through molecular interaction with the key targets above.

## Data Availability

The GSE70362 dataset used in this study can be found in the Gene Expression Omnibus (http://www.nicb.nlm.nih.gov/geo/).

## Abbreviations

IVDD: Intervertebral disc degeneration
IVD: Intervertebral disc
AF: Annulus fibrosus
NP: Nucleus pulposus
ECM: Extracellular matrix
BMs: Basement membranes
PPI: Protein-to-protein interaction
GO: Gene ontology
GEO: Gene Expression Omnibus
DSigDB: Drug Signature database
OMIM: Online Mendelian Inheritance in Man
STRING: Search Tool for the Retrieval of Interacting Genes
MCODE: molecular complex detection technology
BP: Biological processes
GAG: Glycosaminoglycan
ADAMTS: Metalloprotease within thrombospondin motifs
DCN: decorin
FBN1: Fibrillin-1
HSPG2: Heparin sulfate proteoglycan 2
